# Impact of gender on the prognosis of carotid body tumor after surgical resection

**DOI:** 10.1186/s40463-021-00540-y

**Published:** 2021-09-27

**Authors:** Huanrui Hu, Yuwei Xiang, Bin Huang, Ding Yuan, Yi Yang, Jichun Zhao

**Affiliations:** 1 Department of Vascular Surgery and National Clinical Research Center for Geriatrics, West China Hospital of Sichuan University, 37 Guo Xue Alley, Chengdu, 0086-610041 Sichuan Province China; 2 West China-Washington Mitochondria and Metabolism Center, West China Hospital of Sichuan University, 37 Guo Xue Alley, Chengdu, 0086-610041 Sichuan Province China

**Keywords:** Carotid body tumor, Gender, Surgery outcomes, Prognosis

## Abstract

**Background:**

Carotid body tumors (CBTs) are rare neuroendocrine neoplasms, but the prognosis of patients with resected CBTs has seldom been elucidated. This study was conducted to investigate the association between variables, especially sex, and the prognosis of carotid body tumor resection.

**Methods:**

This was a large-volume single-center retrospective cohort study. Patients who were diagnosed with CBTs between 2009 and 2020 at our center were analyzed retrospectively. Their preoperative, surgical, and follow-up data were collected, and the association between variables and outcomes of CBT resection was assessed by correlation analysis, multivariate logistic regression, and multivariate Cox regression as appropriate.

**Results:**

A total of 326 patients (66.6% were females) were included. Males developed larger CBTs than females (4.3 ± 1.8 cm vs. 3.8 ± 1.4 cm, *P* = .003). Males were more likely to develop succinate dehydrogenase B (SDHB) mutations (*P* = .019) and had worse relapse-free survival rates (*P* = .024). Although tumor size and Shamblin classification had positive relationships with neurological complications and intraoperative blood loss, they did not affect the overall survival rate of patients, which was only influenced by remote metastasis (*P* = .007) and local recurrence (*P* = .008).

**Conclusions:**

Compared to females, males with CBT resection were found to have more SDHB mutations and worse relapse-free survival rates, which may lead to the deterioration of prognosis. Tumor size and Shamblin classification cannot predict the overall survival rate of patients with excised CBTs.

**Graphical abstract:**

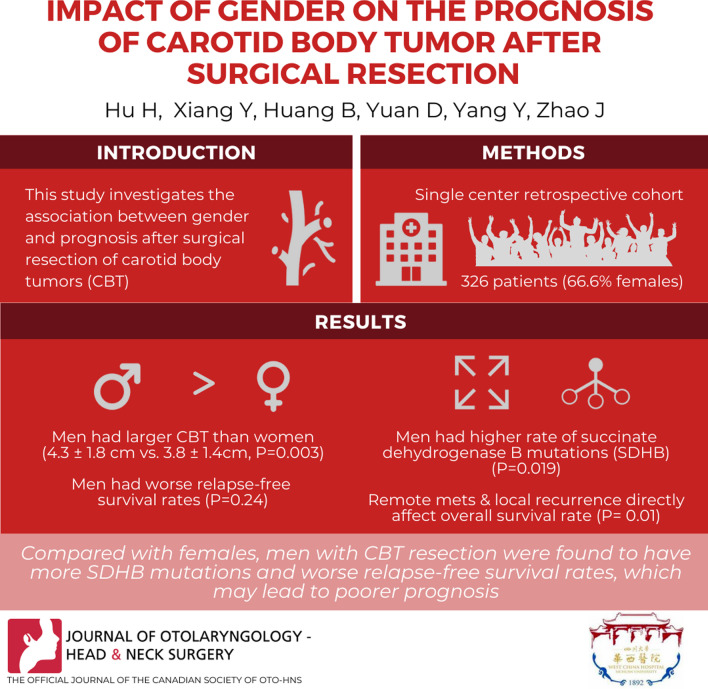

## Background

Carotid body tumors (CBTs) are rare neuroendocrine neoplasms [1] typically located at the bifurcation of the carotid artery. CBTs have been pathologically classified as paragangliomas [2]. Unlike other types of tumors, malignant paragangliomas cannot be simply distinguished by certain markers or tests [3], and they are normally identified by the presence of distant metastasis [4]. Thus, although CBTs grow slowly, all of them are believed to have the potential for malignant transformation [5]. Currently, the primary treatment for CBTs is surgery, which results in many operative complications [6, 7]. However, the prognosis of patients with CBT resection has seldom been elucidated. We conducted this study to investigate the predictors of surgical outcomes and the prognosis of CBT resection. In addition, we noticed that more females had CBTs than males in previous studies [8]. Thus, we speculated that sex may influence the prognosis of CBTs. In this study, we focused on the relationship between sex and the prognosis of CBT resection, which has not been investigated before.

## Patients and methods

### Patients

This study was approved by the Ethics Committee of Sichuan University. All patients who were diagnosed with CBTs between 2009 and 2020 at West China Hospital were included. Patients were identified by their admitting diagnosis and admission code. Each record of patients was reviewed to collect their demographic, preoperative, perioperative, and postoperative information. CBT dimensions were measured from preoperative computed tomography (CT) scans or magnetic resonance imaging (MRI). Patients who 1) were managed nonoperatively; 2) had incomplete imaging or operative records; 3) were not confirmed by pathological examination; and 4) underwent a second procedure for bilateral CBT were excluded.

Surgical outcomes were recorded, including intraoperative estimated blood loss (EBL) and neurological injuries that included temporary sensory disturbances and permanent injuries of cranial nerves. Patients were investigated at 6 and 12 months and yearly after surgery. Neurological function was evaluated first, and neurologists were invited for consultation if necessary. Then, CT scans were performed to detect local recurrence and distant metastasis, and follow-up data were recorded according to a standardized format. The primary endpoint was the overall survival rate, and the secondary endpoint was the relapse-free survival (RFS) rate, defined as the length of time between CBT resection and the development of local recurrence or distant metastasis.

### Surgical procedure

Nine experienced surgeons performed CBT resection surgery with a standardized protocol. Briefly, all CBTs were evaluated by imaging examination before operations. No preoperative embolization was performed for any of the patients. If CBTs were graded as Shamblin I, resection was performed directly without interfering with the carotid artery. If CBTs were graded as Shamblin II or III, which are likely to invade carotid arteries, assessment for autologous great saphenous vein (GSV) transplantation was performed. If the patients had bilateral CBTs, their Shamblin grade was recorded according to the most severe side. When patients developed phlebothrombosis in the lower limbs or the diameter of proximal GSVs was smaller than 3 mm, Gore-Tex artificial grafts were adopted for carotid artery reconstruction. Invaded vessels were resected together with tumors, and reconstruction was conducted by patch, bypass, or direct anastomosis according to the size of the lesion. If the carotid sinus was invaded and needed resection, the graft was placed from the common carotid artery (CCA) to the internal carotid artery (ICA), and the external carotid artery (ECA) was ligated. In cases in which the carotid arteries requires clamping, the ICA cannot be blocked for more than 3 min. If more time is required for vessel reconstruction or tumor resection, the reflux pressure of the ICA should be measured after CCA and ECA are clamped. When reflux pressure reaches below 30 mmHg, a shunt is required from the CCA to the ICA to maintain the necessary blood flow for brain function. Intraoperative nerve injuries were repaired if possible and were recorded in operative documents. Estimated blood loss was recorded by anesthetists involved in the operations. Complete resection was confirmed by histological examination of the specimen with particular reference to the tumor margin. The biopsy was checked by two different pathologists.

### Statistics

Continuous variables are presented as the mean ± standard deviation (SD), and cohort characteristics are presented as absolute numbers with percentages in parentheses. Follow-up and survival intervals are presented as medians with range values. Data were collected using Microsoft Excel and analyzed with the SPSS 24.0 statistical package for Windows and GraphPad Prism 6. Differences between two groups of continuous variables were analyzed by a t-test or the Mann–Whitney U test according to their distributions. Dichotomous outcomes and clinical variables were analyzed by Fisher’s exact test or the chi-square test. For estimated blood loss, correlation statistics were calculated using Pearson’s or Spearman’s correlation for continuous or binary variables, respectively. Multivariate logistic regression was used to identify the independent risk factors for any nerve injury. The association between variables and follow-up outcomes was assessed by the Kaplan–Meier method with censored survival and multivariate Cox regression. Statistical significance was defined as *P* < 0.05.

## Results

### Patient demographics and tumor characteristics

A total of 326 patients (217 females) were included in this study. Among them, 45 patients had bilateral CBTs. The average age of these patients was 47.5 years, ranging from 15 to 81 years. The most common presenting symptom was a neck mass (n = 209); the next most common presentation was symptoms induced by cranial nerve compression or invasion (Horner syndrome, hoarseness, and tongue paralysis). Five patients (one male) developed vagal tumors simultaneously, and these tumors were resected together with CBTs. Patients were divided into two groups according to their sex. Other than tumor size and SDHB gene mutation, there was no significant difference in patients’ clinical characteristics between the two groups. Compared with females, males developed larger CBTs (4.3 ± 1.8 cm vs. 3.8 ± 1.4 cm, *P* = 0.003) and had a higher rate of SDHB mutation (23.5% vs. 8.5%, *P* = 0.019). Detailed patient demographics and tumor characteristics are listed in Table 1.

**Table 1:** Patient Demographics and Tumor Characteristics

Characteristic	Male (n = 109)	Female (n = 217)	*P* value
Total tumor number	126	252	
Tumor size (cm)	4.3 ± 1.8	3.8 ± 1.4	.003
Age at diagnosis (years)	47.6 ± 13.9	47.3 ± 12.3	.856
Symptoms			
Neck mass	72 (66.1)	137 (63.1)	.627
Dysphagia	5 (4.6)	7 (3.2)	.544
Tinnitus	1 (0.9)	6 (2.8)	.431
Hoarseness	8 (7.3)	9 (4.1)	.290
Horner syndrome	3 (2.8)	10 (4.6)	.555
Tongue paralysis	11 (10.1)	18 (8.3)	.681
Tumor location			
Left-side	40	96	.193
Right-side	52	100	.782
Bilateral	17	28	.506
Shamblin classification			
I	16 (14.6)	41 (18.9)	.284
II	43 (39.4)	81 (37.3)	.890
III	50 (45.9)	95 (43.8)	.928
SDHB gene screen	51 (46.8)	106 (48.8)	.726
Mutation positive	12 (23.5)	9 (8.5)	.019
Local invasion	27 (24.8)	51 (23.6)	.800

Data are presented as n (%) or mean ± SD unless stated otherwise

### Surgical outcomes

All CBTs were resected completely, and only one patient died of cerebral hernia caused by severe cerebral infarction on the 21st day after the operation. Based on intraoperative findings, CBTs were classified according to Shamblin grade [9]. As shown in Table 1, there was no significant difference in the Shamblin grade classification between the two groups. Since 2015, 157 patients (67.5% female) had been tested for succinate dehydrogenase B (SDHB) mutations, and more males developed SDHB mutations than females (23.5% vs. 8.5%, *P* = 0.019). As shown in Table 2, the average EBL of males and females was 974.0 ± 763.2 ml and 690.8 ± 583.7 ml, respectively. Two males and five females were reported to have experienced a stroke postoperatively. Among these patients, two females were classified as Shamblin II, and the rest were classified as Shamblin III. All of their carotid arteries were clamped for vascular reconstruction during the operation procedure. The entire invaded section of each artery was replaced by an artificial graft in one patient and an autograft in another. Patches were used to reconstruct the partially excised vessels in the other five patients. Persistent postoperative hypotension, which was defined as systolic blood pressure lower than 90 mmHg for more than 2 h after anesthesia resuscitation, developed in one male patient and three female patients who were administered norepinephrine with a micropump. One patient underwent tracheotomy for laryngeal obstruction. Forty-five males (38.5%) and 58 females (26.7%) had neurological complications caused by nerve injuries or disturbance. However, after correction for confounding factors, no significant difference was observed in EBL and nerve injury between the groups (Tables 3 and 4). EBL and nerve injuries were associated with tumor size and Shamblin grade classification, and smaller CBTs and lower Shamblin grade were associated with fewer nerve injuries and less blood loss; details are listed in Tables 3 and 4.

**Table 2:** Surgical outcomes and Follow-up

Univariate variables	Male (n = 109)	Female (n = 217)	*P* value
EBL (ml)	974.0 ± 763.2	690.8 ± 583.7	.021
Perioperative complications			
Cerebral infarction	2 (1.8)	5 (2.3)	1.000
Postoperative hypotension	1 (0.9)	3 (1.4)	1.000
Laryngeal obstruction	1 (0.9)	0	.334
Nerve injury*			
Any nerve injury	45 (38.5)	58 (26.7)	.008
Facial nerve	7 (6.4)	10 (4.6)	.667
Glossopharyngeal nerve	4 (3.6)	12 (5.5)	.592
Vagus nerve	29 (26.6)	36 (16.6)	.032
Accessory nerve	3 (2.8)	4 (1.8)	.690
Hypoglossal nerve	35 (32.1)	44 (20.2)	.019
Superior laryngeal nerve	3 (2.8)	4 (1.8)	.690
Recurrent laryngeal nerve	1 (0.9)	3 (1.4)	1.000
Multiple nerves injuries	25 (22.9)	31 (14.2)	.051
Perioperative mortality	0	1 (0.5)	1.000
Follow-up (mo)	62.5 ± 33.6	60.5 ± 33.6	.601
Local recurrence	15 (13.8)	12 (5.5)	.011
Distant metastasis	18 (16.5)	17 (7.8)	.096
Stroke	8 (7.3)	15 (6.9)	.554
Permanent nerve injury	18 (16.5)	23 (10.6)	.330
Deceased	7 (6.4)	8 (3.7)	.220
Withdraw	9 (8.3)	14 (6.4)	.548

Data are presented as n (%) or mean ± SD unless stated otherwise

^*^Include Horner syndrome, hoarseness, dysphagia, tongue muscle atrophy, local paralysis, *EBL* Estimated blood loss

**Table 3:** Correlation between variables and estimated blood loss

	Female^#^	Age*	Maximal diameter*	Shamblin grade^#^	SDHB mutation^#^	Preoperative neurological symptoms^#^
Coefficient	− .106	− .026	.612	.461	− .142	.102
*P* value	.226	.767	< .001	< .001	.403	.248

^*^Pearson correlation was used for continuous variables

^#^Spearman correlation was used for binary variables

**Table 4:** Multivariate logistic regression with risk factors for any nerve injury

Variables	OR	95%CI	*P* value
Female	1.00	0.39–2.53	.079
Age	0.99	0.94–1.05	.857
Maximal diameter	1.96	1.67–2.38	.004
Shamblin grade	6.06	2.34–15.65	< .001
SDHB mutation	2.78	0.83–3.33	.420
Preoperative neurological symptoms	1.33	0.30–5.88	.157

*OR* odd ratio; *CI* confidence interval;

### Follow-up

**Fig. 1:**
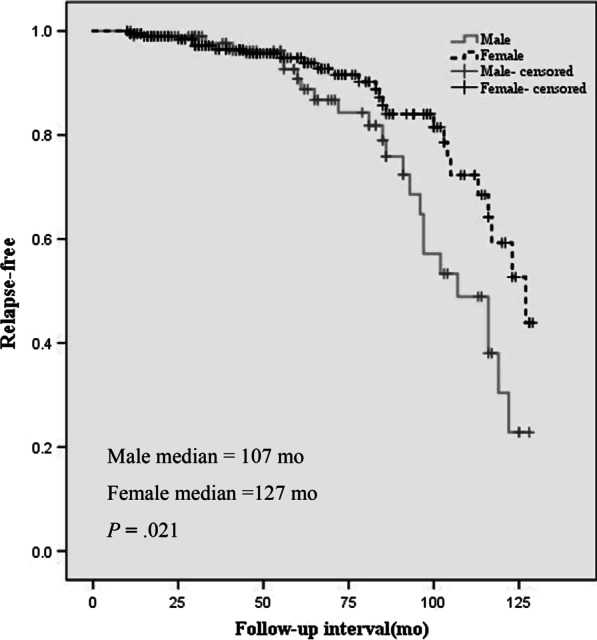
Kaplan–Meier analysis demonstrated males had shorter relapse-free survival (*P* = .021)

The mean follow-up times of males and females were 62.5 and 60.5 months, respectively. The overall survival rate of males was 93.6%, and that of females was 96.3%. Twenty-three patients (9 males) were lost to follow-up. Twelve patients developed both local recurrence and distant metastasis. Although there was no significant difference in their overall survival rate, males (13.8%) developed more local recurrence than females (5.5%) and had a shorter RFS rate. As shown in Fig. 1, 50% of the males had experienced relapse at 107 months, while 50% of the females experienced relapse at 127 months (*P* = 0.021). As listed in Table 5, Cox proportional hazards regression showed that both remote metastasis (HR = 4.01, *P* = 0.019) and local recurrence (HR = 6.46, *P* = 0.010) had a positive relationship with mortality, and both female sex (HR = 0.78, *P* = 0.028) and SDHB mutation (HR = 3.52, *P* = 0.032) had a correlation with the RFS rate.

**Table 5:** Multivariate cox regression between variables and follow-up outcomes

	Deceased (HR, 95% CI)	RFS (HR, 95% CI)
Female	0.13, 0.02–1.51	0.78, 0.50–0.86*
Maximal diameter	1.03, 0.35–3.03	1.05, 0.63–1.75
Shamblin grade	2.52, 0.80–3.15	1.08, 0.38–3.03
SDHB mutation	3.86, 0.33–45.45	3.52, 1.11–11.14*
Remote metastasis	4.01, 1.66–8.28*	
Local recurrence	6.46, 2.30–9.61*	
Stroke	1.62, 0.48–4.04	

*HR* hazard ratio; *CI* confidence interval

^*^P < .05; RFS: relapse free survival

## Discussion

In this study, we analyzed variables associated with surgical, RFS and overall survival outcomes after CBT resection. The relationship between sex and the prognosis of CBTs has not been previously investigated. The most important finding was that male patients were more likely to develop local recurrence and had shorter RFS times after CBT resections. This result suggests that CBTs that develop in males have more aggressive phenotypes. Consistent with the findings of previous studies [10, 11], males with CBTs were more likely to develop SDHB mutations than females. In recent years, studies have revealed that SDHB mutation predicts worse paraganglioma prognosis [12, 13]. With multivariate Cox regression (Table 5), we found that male sex and SDHB mutation were both independent risk factors for RFS. Males with SDHB mutations should be more closely followed after CBT resection, and molecular targeted therapy might help to improve the RFS time for individuals with SDHB mutations [14]. However, it is worth noting that only a fraction of our cohort had SDH testing available, and the nontested patients could not be SDH status; thus, the association between SDHB mutation and the RFS rate needs further investigation. Most likely owing to the relatively short follow-up period of this study, there was no significant difference in overall survival rates between males and females, and the overall survival rate was influenced only by local recurrence and remote metastasis. Similar to other studies [5] of large cohorts, females comprised the majority of CBT patients in our hospital. However, considering the rarity of CBTs, epidemiologic studies based on a large population are necessary to investigate the association between CBT morbidity and sex.

Males had more EBL and neurological complications after surgery in this cohort. However, these results may result from the larger CBT size in males. Through multivariate analysis, we confirmed that tumor size and Shamblin grade classification were independent predictors of surgical outcomes. However, these parameters did not affect overall survival or RFS rates. Previous studies [5, 10, 11] have demonstrated that tumor size is positively associated with neurological complications and blood loss during surgery. Shamblin classification was deemed to be another predictor of complications of CBT resection, supporting the theory that the resection of extensively invasive CBT leads to more complications. Shamblin classification can also be utilized to evaluate the potential need for vascular reconstruction. In a recent study [5], tumor distance to the base of skull (DTBOS) and tumor volume were identified as two new predictors of surgical outcomes, and they can be used in combination with tumor size and Shamblin grade classification to better predict bleeding and cranial nerve injury risk. Simultaneous utilization of the aforementioned predictors can be helpful for predicting surgical risks preoperatively.

The surgery performed for CBTs was safe. Postoperative mortality in this cohort was low, and only one patient died within 30 days after surgery. Complications caused by cranial nerve injuries were the most common complications of CBT resection [9]. To date, there is no available method that can be used to avoid these neurological complications. In recent years, preoperative embolization has been commonly used as a new technique for CBT treatment. However, it was unknown whether preoperative embolization was truly effective for reducing intraoperative blood loss [8, 15, 16]. There was no reliable evidence proving that preoperative embolization can improve the surgical outcomes of CBT resection.

There were some limitations to this study. The follow-up interval was not long enough to analyze the influence of sex on overall survival rates after surgery. Additionally, blood loss during surgery cannot be precisely measured. There were no criteria for categorizing postoperative neurological complications according to their severity.

## Conclusion

In this study, we found that males who underwent CBT resection had more SDHB mutations and higher local recurrence rates than females. Patients with SDHB mutation had a shorter RFS time. The overall survival rate of patients was only affected by local recurrence and distant metastasis. These findings provide a more complete picture of the risks and prognoses of CBT resection. Considering the close association between the RFS rate of CBTs and the life expectancy of patients, lifelong surveillance is recommended.

## Data Availability

The datasets used and/or analyzed during the current study are available from the corresponding author on reasonable request.

## Abbreviations

CBT: Carotid body tumor
SDHB: Succinate dehydrogenase B
CT: Computed tomography
MRI: Magnetic resonance imaging
EBL: Estimated blood loss
RFS: Relapse-free survival
GSV: Great saphenous veins
CCA: Common carotid artery
ICA: Internal carotid artery
ECA: External carotid artery
SD: Standard deviation
